# Absolute and Relative Morbidity Burdens Attributable to Various Illnesses and Injuries Among Non-Service Member Beneficiaries of the Military Health System, 2024

**Published:** 2025-09-20

The Military Health System (MHS), a global, integrated health delivery system, is tasked with ensuring the medical readiness of the U.S. Armed Forces while fulfilling the individual health care needs of eligible military personnel and their dependents. ^1^ The MHS network comprises military hospitals and clinics worldwide (collectively called the “direct care system”), complemented by programs that enable care in the private sector through the TRICARE insurance program. While the first mission of the MHS enables the National Defense Strategy through a medically ready force, the inter-related mission to provide a medical benefit commensurate with the service and sacrifice of the U.S. Armed Forces extended TRICARE eligibility to approximately 9.4 million beneficiaries in fiscal year 2024. ^2 , 3^

MHS beneficiaries are a diverse and heterogeneous population of service members, military retirees, and family members from all branches of military service under the authority of the Department of Defense. ^2^ Accordingly, each beneficiary category presents its own demographic, enrollment, and health care provision patterns. In fiscal years 2024 through 2029, the Military Health System Strategy prioritizes stability for the direct care system through a dedicated strategic objective to “attract and reattract beneficiaries to military treatment facilities, to improve efficiency and enrich clinical experience for the Ready Medical Force, and consciously fulfill the promise our nation makes to care for our beneficiaries.” ^3^

Beneficiaries enrolled in TRICARE, including many family members of service members and eligible retirees (primarily those aged 64 years and younger), may receive care at fixed military hospitals and clinics, or from private sector health care facilities that supplement direct military medical care. An important element of beneficiary care is the transition from TRI-CARE to Medicare. Once an individual reaches age 65, and becomes eligible for Medicare, TRICARE eligibility ends. If individuals enroll in Medicare, they receive a Medicare gap insurance, known as TRI-CARE for Life (TFL), funded through mechanisms outside of the Defense Health Program. While Medicare-eligible individuals remain eligible for direct care at military medical facilities, such care is contingent upon resource availability. Consequently, distribution of health care burden estimates should be considered in relation to beneficiary age category and source of care when interpreting health care provision data among MHS beneficiaries.

This report represents an updated summary of health care burdens among non-service member MHS beneficiaries during calendar year 2024. Health care burdens were quantified using a classification system derived from the Global Burden of Disease (GBD) Study, ^4 - 7^ in combination with diagnostic groupings from the International Classification of Diseases, 10th Revision, Clinical Modification (ICD-10-CM) chapter-based system for categorizing hospitalizations and ambulatory visits. This report presents stratified estimates for 4 age groups of health care recipients, with Medicare-eligible beneficiaries (over age 65 years) considered separately, as most of their care is provided and paid by non-MHS resources.

**What are the new findings?**

In 2024, mental health disorders accounted for the largest proportions of morbidity and health care burdens that affected the pediatric and younger adult age groups of non-service member Military Health System beneficiaries. Among adult beneficiaries older than age 45, musculoskeletal diseases was the leading diagnostic category for medical encounters. While provision of care from purchased care reimbursements or military medical facilities varied by age category, a majority of non-service member beneficiaries received care exclusively from private sector facilities.

**What is the impact on readiness and force health protection?**

Military Health System beneficiaries are a diverse, heterogeneous population of service members, retirees, and family members from all branches of military service under the U.S. Department of Defense. Each category of beneficiaries presents its own demographic, enrollment, and health care use patterns. The 2024-2029 Military Health System Strategy calls to attract and re-attract beneficiaries to military medical facilities, to improve efficiency, enrich the clinical experience for the ready medical force, and consciously fulfill the nation's promise to care for Military Health System beneficiaries. Routinely documented and reported trends in health care use and diagnostic patterns can help senior leaders improve resource allocation within the Military Health System to maximize efficiency, medical readiness, and the readiness of the medical forces.

## Methods

The surveillance population included all non-service member MHS beneficiaries who had at least 1 hospitalization or out-patient medical encounter from January 1 through December 31, 2024, with either a military hospital, clinic or health care provider, or through a private sector facility or provider (if reimbursed through TRI-CARE or through Medicare with a co-payment by TFL). All inpatient and outpatient medical encounters for this analysis were summarized according to the primary (i.e., first-listed) International Classification of Diseases, 10th Revision (ICD-10) codes that indicate the natures of illnesses or injuries (A00–T88). Nearly all records of encounters with first-listed diagnoses coded with ‘Z’ (care other than for a current illness or injury, e.g., general medical examinations, after care, vaccinations) or ‘V’, ‘W’, ‘X’, or ‘Y’ (indicators of the external causes but not the natures of injuries) were excluded from the analysis; encounters with a code of Z37 (“outcome of delivery”) in the primary position were retained.

For summary purposes, all illness and injury-specific diagnoses (as defined by ICD-10) were grouped into 157 burden of disease-related conditions and 25 major morbidity categories, based upon a modified version of the classification system developed for the Global Burden of Disease Study. This year, 4 new diagnostic groups were added: pain in foot, chronic rhinitis, neoplasm of uncertain behavior of skin, and disorder of the pituitary gland. The methodology for summarizing absolute and relative morbidity has been used annually since 2014 and is described else-where. ^8^ Results were stratified by source of health care (direct care, i.e., military hospitals and clinics vs. non-direct care, i.e., private sector medical facilities) and by age group (0-17 years, 18-44 years, 45-64 years, 65 years and older). For analysis of morbidity burdens within the youngest age group, developmental disorders were included in the general category of mental health disorders.

## Results

In 2024, the population of non-service member MHS care recipients included more female (56.8%) than male (43.2%) beneficiaries. Adults aged 65 years and older accounted for the highest number of individuals receiving health care (n=2.04 million, 33.0%), followed by pediatric beneficiaries aged 17 years and younger (n=1.46 million, 23.7%), adults ages 18-44 years (n=1.37 million, 22.2%), and older adults ages 45-64 years (n=1.30 million, 21.0%) **(Table 1)** .

**TABLE 1.** Medical Encounters ^a^ , Individuals Affected ^b^ and Hospital Bed Days, by Source of Care and Age Group, Non-Service Member MHS Beneficiaries, 2024

	Medical Encounters		Individuals Affected		Hospital Bed Days		Medical Encounters per Individual Affected
	No.	%	No.	%	No.	%	
All non-service member beneficiaries	90,357,451	—	6,180,903	—	6,261,731	—	14.6
Source of care							
Direct care only	7,289,625	8.1	502,574	8.1	275,933	4.4	14.5
Outsourced care only ^c^	83,067,826	91.9	4,701,644	76.1	5,985,798	95.6	17.7
Direct and outsourced care ^d^	N/A	N/A	976,685	15.8	N/A	N/A	N/A
Age group, y							
0–17	13,594,874	15.0	1,464,102	23.7	507,922	8.1	9.3
18–44	13,423,894	14.9	1,373,626	22.2	615,156	9.8	9.8
45–64	17,320,065	19.2	1,300,764	21.0	781,706	12.5	13.3
65+	46,018,598	50.9	2,042,408	33.0	4,356,947	69.6	22.5
Unknown	40	0	3	0	0	0	13

Abbreviations: MHS, Military Health System; No., number; N/A, not applicable; y, years.

a Medical encounters include total hospitalizations and ambulatory visits for the condition (with no more than 1 encounter per individual per day per condition).

b Individuals with at least 1 hospitalization or ambulatory visit for the condition.

c Represents encounters or hospital bed days received under purchased care or care received under Medicare benefit.

d Represents a combination of care received directly at military hospitals or clinics and non-military medical facilities.

A total of 6,180,903 non-service member MHS beneficiaries had 90,357,451 recorded medical encounters in 2024. Over half (50.9%) of these medical encounters were among 2,042,408 MHS beneficiaries aged 65 years or older **(Table 1)** . Provision of care for this age group was almost exclusively outsourced, with 91.0% of individuals age 65 years or older having medical encounters or hospital bed days documented only from purchased care reimbursements at private sector facilities **(Table 2)** .

**TABLE 2.** Individuals Affected ^a^ , by Age Group and Source of Care, Non-Service Member MHS Beneficiaries, 2024

	Direct Care Only		Outsourced Care Only ^b^		Direct and Outsourced Care ^c^		Total
Age group, y	No.	%	No.	%	No.	%	
0–17	165,998	11.3	1,008,310	68.9	289,794	19.8	1,464,102
18–44	193,390	14.1	900,341	65.5	279,895	20.4	1,373,626
45–64	101,318	7.8	934,190	71.8	265,256	20.4	1,300,764
65+	41,867	2.0	1,858,803	91.0	141,738	6.9	2,042,408
Unknown	1		0		2		3

Abbreviations: MHS, Military Health System; y, years; No., number.

a Individuals with at least 1 hospitalization or ambulatory visit for the condition.

b Represents encounters or hospital bed days received under purchased care or care received under Medicare benefit.

c Represents a combination of care received directly at military hospitals or clinics and non-military medical facilities.

Among TRICARE-eligible beneficiaries (under age 65 years), provision of care was also primarily exclusively from outsourced care. Adults ages 18-44 years received approximately one-third of their care exclusively from military clinics and hospitals (14.1%) or a combination of direct and outsourced care (20.4%) **(Table 2)** . The 3 most frequent morbidity-related categories accounting for the most medical encounters among TRICARE-eligible beneficiaries included mental health disorders, signs or symptoms of ill-defined conditions, and injury **(Figure 1a)** . Mental health disorders also represented the leading category for hospital bed days among beneficiaries under age 65 years, followed by maternal conditions **(Figure 1b)** .

**FIGURE 1a.**
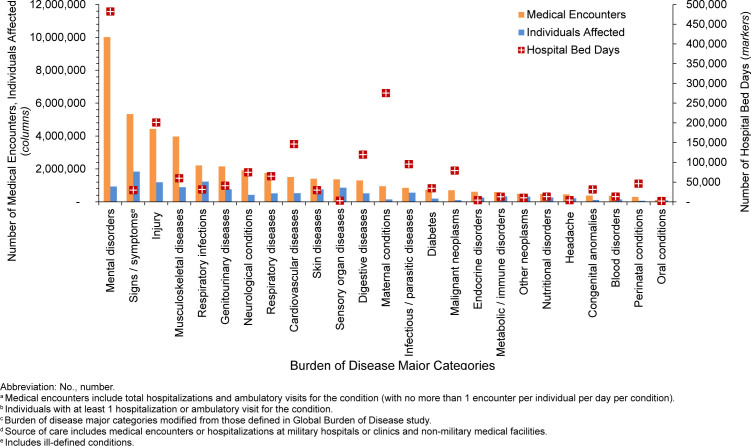
Numbers of Medical Encounters ^a^ , Individuals Affected ^b^ and Hospital Bed Days, by Burden of Disease Major Category ^c^ , Non-Service Member MHS Beneficiaries ^d^ Under Age 65 Years, 2024

**FIGURE 1b.**
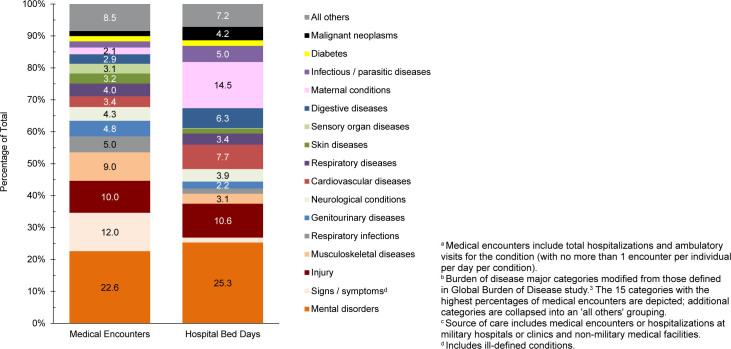
Percentages of Medical Encounters ^a^ and Hospital Bed Days, by Burden of Disease Major Category ^b^ , Non-Service Member MHS Beneficiaries ^c^ Under Age 65 Years, 2024

### Pediatric beneficiaries under age 18 years

Pediatric patients accounted for 15.0% of all medical encounters, 23.7% of all individuals affected, and 8.1% of all hospital bed days among non-service member MHS beneficiaries in 2024 **(Table 1)** . On average, each pediatric beneficiary had 9.3 medical encounters during the year. Provision of care for pediatric patients was primarily through exclusive use of purchased care reimbursement in private settings (68.9%), followed by a combination of direct and outsourced care (19.8%). Only 11.3% of pediatric patients received all medical encounters or hospital bed days from direct MHS care **(Table 2)** .

In 2024, mental health disorders represented the largest burden of disease among pediatric beneficiary medical encounters (38.7%, n=5,260,830) and contributed to the highest number of hospital bed stays (58.1%, n=295,259) **(Figures 2a** , **2b)** . On average, pediatric beneficiaries affected by a mental health disorder experienced 15.9 medical encounters during the year specifically related to this morbidity category (data not shown). More than two-thirds (69.2%) of all medical encounters for mental health disorders among pediatric beneficiaries were attributed to 3 groups of disorders: autistic disorder and pervasive developmental disorders (33.8%), developmental disorders of speech and language (24.4%), and attention-deficit hyperactivity disorders (11.0%) **(Figure 2c)** . Pediatric patients affected by an autistic disorder had, on average, 41.2 autism-related encounters per individual (data not shown). Despite the high numbers of encounters associated with these 3 categories of mental health disorders, over two-thirds (68.6%) of hospital bed days related to mental health disorders were attributable to mood disorders. Among all mood disorder-related bed days, over 50% were attributed to 2 diagnostic categories: recurrent severe major depressive disorder without psychotic features (30.6%, ICD10: F332) and disruptive mood dysregulation disorder (28.5%, ICD10: F3481) (data not shown).

**FIGURE 2a.**
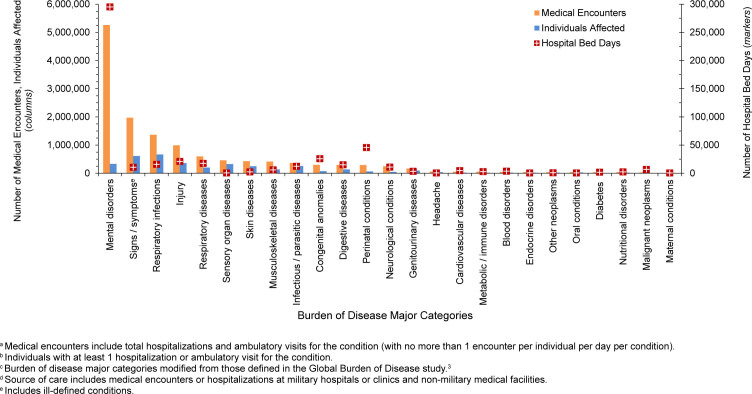
Medical Encounters ^a^ , Individuals Affected ^b^ and Hospital Bed Days, by Burden of Disease Major Category ^c^ , Pediatric Non-Service Member MHS Beneficiaries ^d^ , Ages 0–17 Years, 2024

**FIGURE 2b.**
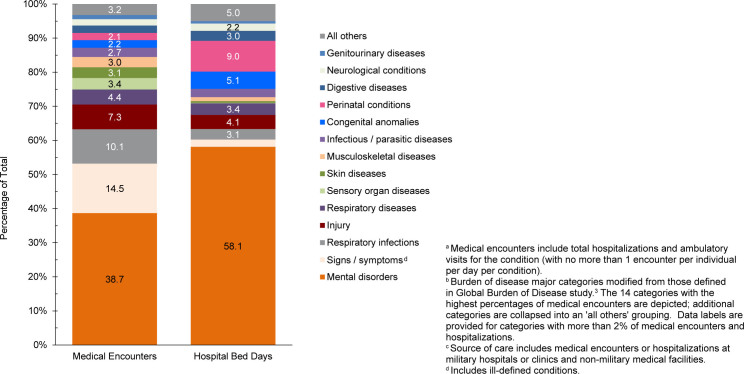
Percentages of Medical Encounters ^a^ and Hospital Bed Days, by Burden of Disease Category ^b^ , Pediatric Non-Service Member MHS Beneficiaries ^c^ , Ages 0–17 Years, 2024

**FIGURE 2c.**
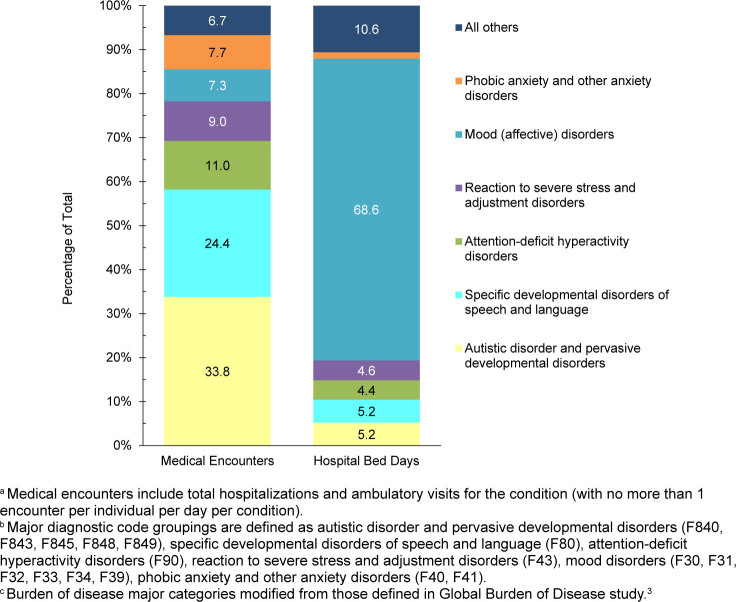
Percentages of Medical Encounters ^a^ and Hospital Bed Days for Major Diagnostic Code Groupings ^b^ Under the Mental Health Disorder Burden of Disease Category ^c^ , Pediatric Non-Service Member MHS Beneficiaries, Ages 0–17 Years, 2024

Perinatal conditions, or medical issues occurring within 1 year of birth, accounted for the second highest number of hospital bed days (n=45,612, 9.0%) in 2024 among pediatric beneficiaries, after mental health disorders **(Figures 2a** , **2b)** . Pediatric beneficiaries affected by malignant neoplasms had, on average, 12.6 neoplasm-related encounters per individual. The highest numbers of malignant neoplasm-related encounters and hospital bed days were attributable to leukemias (data not shown).

Respiratory infections (including upper and lower respiratory infections and otitis media) accounted for more medical encounters among pediatric beneficiaries (10.1%) compared to any older age group of beneficiaries **(Figures 2b** , **3b** , **4b** , **5b)** .

**FIGURE 3a.**
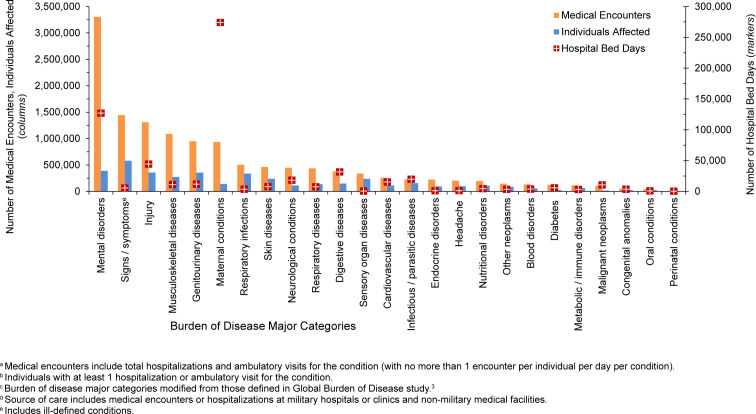
Medical Encounters ^a^ , Individuals Affected ^b^ and Hospital Bed Days, by Burden of Disease Major Category ^c^ , Non-Service Member MHS Beneficiaries ^d^ , Ages 18–44 Years, 2024

**FIGURE 3b.**
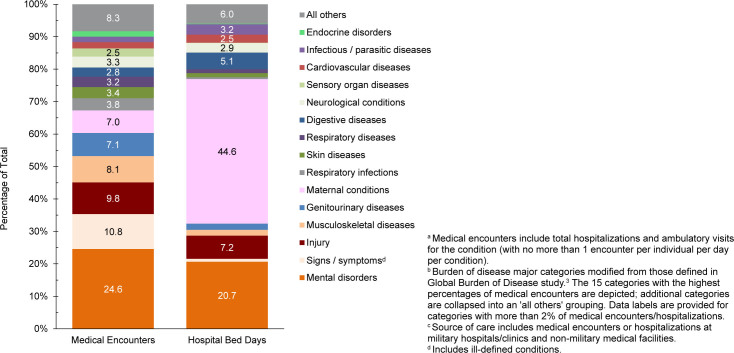
Percentages of Medical Encounters ^a^ and Hospital Bed Days, by Burden of Disease Major Category ^b^ , Non-Service Member MHS Beneficiaries ^c^ , Ages 18–44 Years, 2024

**FIGURE 4a.**
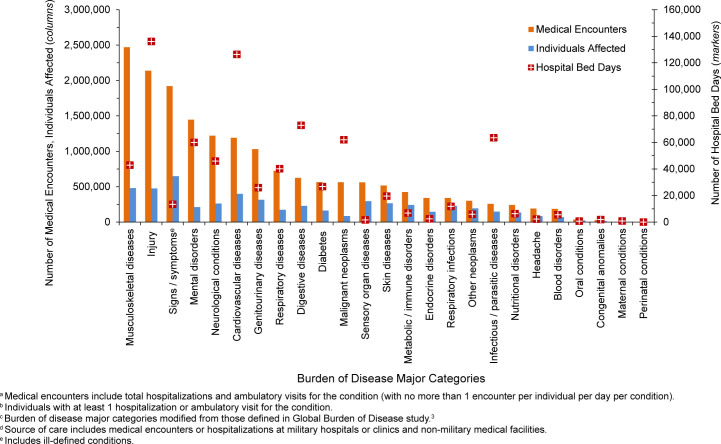
Medical Encounters ^a^ , Individuals Affected ^b^ and Hospital Bed Days, by Burden of Disease Major Category ^c^ , Non-Service Member MHS Beneficiaries ^d^ , Ages 45–64 Years, 2024

**FIGURE 4b.**
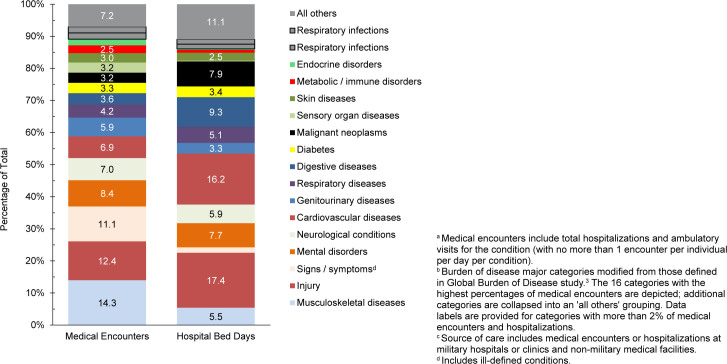
Percentages of Medical Encounters ^a^ and Hospital Bed Days, by Burden of Disease Major Category ^b^ , Non-Service Member MHS Beneficiaries ^c^ , Ages 45–64 Years, 2024

**FIGURE 5a:**
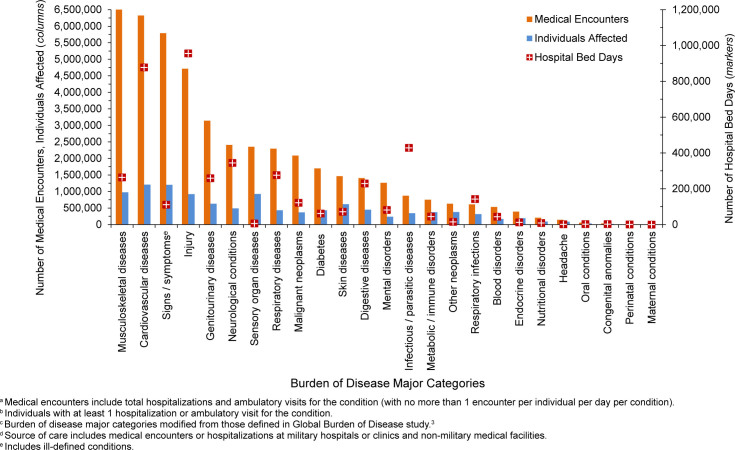
Medical Encounters ^a^ , Individuals Affected ^b^ and Hospital Bed Days by Burden of Disease Major Category ^c^ , Non-Service Member MHS Beneficiaries ^d^ , Age 65 Years or Older, 2024

**FIGURE 5b.**
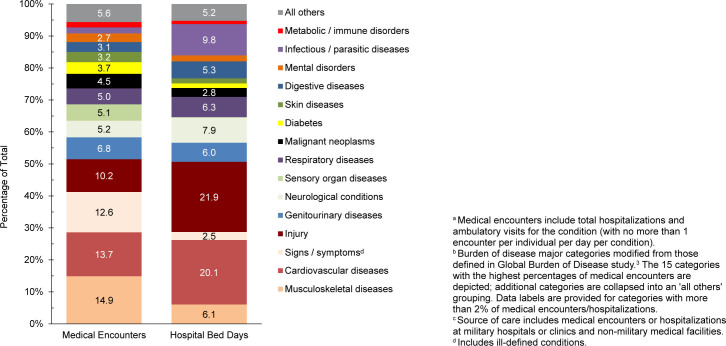
Percentages of Medical Encounters ^a^ and Hospital Bed Days, by Burden of Disease Major Category ^b^ , Non-Service Member MHS Beneficiaries, Age 65 Years or Older ^c^ , 2024

### Beneficiaries ages 18–44 years

Non-service member beneficiaries ages 18-44 years accounted for 14.9% of all medical encounters, 22.2% of all individuals affected, and 9.8% of hospital bed days in 2024 **(Table 1)** . On average, each individual aged 18-44 years affected with an illness or injury (of any cause) had 9.8 medical encounters during the year. Provision of care for beneficiaries ages 18-44 was primarily through exclusive use of purchased care reimbursement in private settings (65.5%), followed by a combination of direct and outsourced care (20.4%). Only 14.1% of beneficiaries ages 18-44 years received all medical encounters or hospital bed days from direct MHS care **(Table 2)** .

Mental health disorders accounted for the most medical encounters (n=3,304,305, 24.6%) among adult MHS beneficiaries ages 18-44 years in 2024 **(Figures 3a** , **3b)** , also representing over one-fifth (20.7%) of total hospital bed days, and, on average, 8.5 mental health disorder-related encounters per individual. Anxiety disorders (35.9%), mood disorders (29.2%), and adjustment disorders (14.8%) accounted for over three-quarters (79.9%) of all medical mental health disorder encounters (data not shown). Mood and substance abuse disorders accounted for over three-quarters (47.1% and 28.6%, respectively) of hospital bed days for mental health disorders.

Maternal conditions accounted for more than two-fifths (n=274,180, 44.6%) of all hospital bed days among adults ages 18-44 years, as well as, on average, 6.7 medical encounters per affected individual **(Figures 3a** , **3b)** . Of the 274,180 hospital bed days for maternal conditions, 62.4% were attributed to pregnancy complications and 20.2% to infant deliveries (data not shown).

Malignant neoplasms, as a diagnostic group, resulted in 6.9 encounters, on average, per individual in 2024. Of the 104,672 medical encounters for malignant neoplasms among adults ages 18-44 years, 32.8% were attributed to malignant neoplasm of the breast (data not shown).

### Beneficiaries ages 45–64 years

Non-service member beneficiaries ages 45-64 years constituted approximately one-fifth (19.2%) of all medical encounters, 21.0% of all individuals affected, and 12.5% of hospital bed days in 2024 **(Table 1)** . Each affected individual aged 45-64 years had, on average, 13.3 medical encounters during the year. Provision of care for beneficiaries ages 45-64 years was primarily through exclusive use of purchased care reimbursement in private settings (71.8%), followed by a combination of direct and outsourced care (20.4%). Only 7.8% of beneficiaries ages 45-64 years received all medical encounters or hospital bed days from direct MHS care **(Table 2)** .

Of all morbidity-related categories, musculoskeletal diseases accounted for the most medical encounters (n=2,469,102, 14.3%) among older adult beneficiaries ages 45-64 years **(Figures 4a** , **4b)** ; back problems accounted for 41.9% of these musculoskeletal disease-related encounters (data not shown). Injury represented the highest proportion of hospital bed days (17.4%), second to cardiovascular disease (16.2%) among adults ages 45-64 years (data not shown). Digestive diseases (9.3%) and malignant neoplasms (7.9%) accounted for larger percentages of total hospital bed days among beneficiaries of this age group, compared to other age groups.

Malignant neoplasm of the breast represented the leading cause of neoplasm-related encounters (25.9%) in adult beneficiaries ages 45-64 years (data not shown).

### Medicare-eligible beneficiaries, ages 65 and older

Non-service member beneficiaries aged 65 years and older accounted for the most medical encounters (50.9%) and more than 2.3 times the number of hospital bed days in 2024 than all other age groups combined. On average, each affected individual in this age group had 22.5 medical encounters during the year **(Table 1)** . The provision of care for Medicare-eligible beneficiaries ages 65 and older was primarily through exclusive use of purchased care reimbursement in private settings (91.0%); only 2.1% received all medical encounters or hospital bed days from direct MHS care **(Table 2)** .

Musculoskeletal diseases (n=6,856,411, 14.9%) and cardiovascular diseases (n=6,323,595, 13.7%) together represented the leading causes for medical encounters among beneficiaries aged 65 years or older, while injury (n=955,546, 21.9%) and cardiovascular diseases (877,678 days, 20.1%) were the leading diagnostic categories for hospital bed days **(Figures 5a** , **5b)** . Back problems accounted for a little more than one-third (35.2%) of all musculoskeletal disease-related medical encounters (data not shown).

## Discussion

This report documents the over-all health care burden of disease among non-service member MHS beneficiaries received through direct care at military hospitals and clinics, in addition to purchased care reimbursements from private sector facilities. In 2024, a substantial majority of non-service member MHS beneficiaries received medical care exclusively at private sector facilities, as only 8.1% of all ambulatory encounters and 4.4% of hospital bed days in 2024 were from direct care at military medical facilities.

The National Ambulatory Medical Care Survey of 2019 documented a substantially lower rate of ambulatory visits (3.2 visits per p-yr) ^9^ among the general U.S. population than among non-service member MHS beneficiaries (14.6 visits per p-yr) reported here. This higher rate of ambulatory visits among non-service member beneficiaries compared to national civilian data was observed for all age groups. Since the National Ambulatory Medical Care survey includes uninsured individuals, financial barriers to care may explain a portion of the lower overall use rate among the general U.S. population, while the families of uniformed personnel require more medical procedures in practice, which is reflected in the composition of the most common directly-provided and purchased procedures. ^10 , 11^

As in previous years, mental health disorders were the leading cause for medical encounters within the pediatric (0-17 years) and young adult (18-44 years) beneficiaries age groups, although the proportion of medical encounters attributed to mental health disorders was markedly lower among young adult (24.6%) than pediatric (38.7%) beneficiaries. Developmental disorders were a significant factor for pediatric beneficiary health care, with almost 70% of medical encounters for mental health disorders attributable to autistic disorder and pervasive developmental disorders, specific developmental disorders of speech and language, or attention-deficit hyperactivity disorders.

The leading diagnostic categories for medical encounters and hospitalizations among adult beneficiaries also reflects 2023 data. ^12^ Among adults older than age 45 years, musculoskeletal diseases continue to represent the leading medical encounter diagnostic category. As in prior years, maternal conditions in adult beneficiaries ages 18-44 years accounted for the highest proportion of hospital bed days. Injury and cardiovascular diseases represent the leading diagnostic category for hospitalization among those aged 45 years and older.

When comparing 2023 and 2024 ambulatory encounters (90,192,185 vs. 90,357,451, respectively) and hospital bed days (6,083,009 vs. 6,261,731, respectively) among non-service member MHS beneficiaries, both remained relatively stable. Since this report does not include person-time nor approximate rates, annual comparisons are not proportionate to changes in the numbers of beneficiaries procuring care. While this report aims to describe morbidity-related diagnoses for all MHS beneficiaries, the data are limited to beneficiaries who received care at military hospitals and clinics, or at private sector medical facilities and reimbursed through TRICARE (as primary or secondary insurance) or through Medicare, if TFL was also billed. Certain forms of care provision, such as that paid with other health insurance and not billed to TRICARE, or paid directly by the patient (or family member), are not captured in this report.

The Military Health System Strategy for Fiscal Years 2024-2029 calls for additional capacity, to facilitate the return of patients including non-service member beneficiaries to military hospitals and clinics, improve their access to care, and increase opportunities for sustaining military clinical readiness for medical forces while delivering quality care to beneficiaries. ^1 , 12^ The need to “attract and reattract” beneficiaries to the direct care setting may be reflected in the data throughout this report, which indicate a substantial proportion of medical encounters and hospitalizations for non-service member MHS beneficiaries exclusively from private sector care. Continued evaluation of health care provision and diagnostic patterns may aid senior leaders' allocation of resources for realization of the current MHS strategy and goals.
